# The Role of Right Ventriculo–Arterial Coupling in Symptoms Presentation of Patients with Hypertrophic Cardiomyopathy

**DOI:** 10.3390/jcm12144796

**Published:** 2023-07-20

**Authors:** Andreas Angelopoulos, Evangelos Oikonomou, Alexios S. Antonopoulos, Panagiotis Theofilis, Konstantinos Kalogeras, Paraskevi Papanikolaou, George Lazaros, Gerasimos Siasos, Dimitris Tousoulis, Konstantinos Tsioufis, Charalambos Vlachopoulos

**Affiliations:** 1 Unit for Inherited and Rare Cardiovascular Diseases, 1st Department of Cardiology, Hippokration Hospital, Medical School of National and Kapodistrian University of Athens, 11527 Athens, Greece; andreasosfp7@yahoo.gr (A.A.); antonopoulosal@yahoo.gr (A.S.A.); panos.theofilis@hotmail.com (P.T.); evipapanikolaou215@gmail.com (P.P.); glaz35@hotmail.com (G.L.); drtousoulis@hotmail.com (D.T.); kptsioufis@gmail.com (K.T.); cvlachop@otenet.gr (C.V.); 2 3rd Department of Cardiology, Sotiria Chest Disease Hospital, Medical School of National and Kapodistrian University of Athens, 11527 Athens, Greece; kalogerask@yahoo.gr (K.K.); ger_sias@hotmail.com (G.S.)

**Keywords:** hypertrophic cardiomyopathy, cardiomyopathies, heart failure

## Abstract

Background: Hypertrophic cardiomyopathy (HCM) is the most common inherited cardiomyopathy. The hallmark of HCM is myocardial fibrosis which contributes to heart failure, arrhythmias, and sudden cardiac death (SCD). Objective: To identify the factors implicated in heart failure symptoms and functional capacity of patients with HCM. Methods: In this cohort study, 43 patients with HCM were recruited. According to functional capacity and symptoms presentation, patients were categorized according to New York Heart Association (NYHA) classification, and echocardiographic measurements of left ventricle systolic and diastolic function were conducted. The echocardiographic assessment of right ventriculo–arterial coupling (RVAC) was made by calculating the tricuspid annular peak systolic tissue Doppler velocity (TASV)/estimated RV systolic pressure (RVSP) ratio. Results: Almost half (51%) of our study population present symptoms of heart failure and were categorized as the symptomatic group—NYHA 2 or higher. Maximum LVOT gradient, RVSP, and the ratio of E/e’ were higher in the symptomatic group compared with the asymptomatic group. TASV was lower in the symptomatic group compared with the asymptomatic group (11 ± 1 cm/s vs. 13 ± 2 cm/s, *p* = 0.04). However, there was no difference in other potentially influential factors, such as heart rate or systemic blood pressure. The SCD risk score does not differ between the two studied groups. The RVAC (estimated with the TASV/RVSP ratio) was lower in the symptomatic group compared with the asymptomatic group (0.32 ± 0.09 vs. 0.46 ± 0.11, *p* < 0.001). Conclusion: A low RVAC (as estimated with TASV/RVSP ratio) value could represent an echocardiographic marker of right ventricular–arterial uncoupling in patients with HCM and impaired functional status.

## 1. Introduction

Hypertrophic cardiomyopathy (HCM) is the most common heritable cardiomyopathy, with a prevalence of 1:500 in the general population [1,2]. Diagnosis of the disease can be challenging given its phenotypic heterogeneity and is based on the identification of a left ventricular (LV) wall thickness of ≥15 mm by echocardiography, computed tomography, or cardiac magnetic resonance (CMR) in the absence of secondary causes [2]. One of the hallmarks of HCM is myocardial remodeling, characterized by cardiomyocyte hypertrophy, sarcomeric disarray, and fibrosis [3,4]. Hypertrophied cardiac muscle cells in both the ventricular septum and LV free wall exhibit bizarre shapes, often maintaining intercellular connections with several adjacent cells [3,5]. Many myocytes are arranged in chaotic and disorganized architectural patterns. Myocardial fibrosis contributes to heart failure, arrhythmias, and sudden cardiac death [6,7,8].

Concerning the clinical course of the disease, HCM is generally characterized by sudden death risk due to fatal ventricular arrythmias, and left ventricular outflow tract (LVOT) obstruction causing heart failure due to diastolic dysfunction as a consequence of LV hypertrophy, fibrosis, and stiffness [9,10]. Another aspect of diastolic dysfunction contributing to functional impairment in patients with HCM is left atrium (LA) enlargement, stiffening, and atrial fibrillation [11]. It is of interest that the clinical presentation of patients with HCM varies widely from completely asymptomatic, including patients identified incidentally, to end-stage heart failure. A large proportion of patients with HCM have mild to severe functional status impairment, which is often expressed as dyspnea in exertion and fatigue [12].

For most of the patients with chronic disability from heart failure, the primary cause is LVOT obstruction, which causes markedly elevated left ventricular pressures and secondary mitral regurgitation [13]. Heart failure symptoms may occur or increase in severity at any age, most frequently in mid-life due to LVOT obstruction [14]. Heart failure symptoms due to subaortic obstruction represent a treatable consequence of the disease [14]. Additionally, heart failure in patients with HCM is often accompanied by pulmonary hypertension (PH), diastolic dysfunction, and the absence of an increase in stroke volume with exercise [15]. Moreover, it is known that the ability of the right ventricle (RV) to compensate with the preserved systolic function in the face of an increasing afterload is referred to as maintaining RV-pulmonary artery (PA) coupling [16]. Echocardiographic measures of RV–PA coupling include the ratio of RV longitudinal motion (tricuspid annular plane systolic excursion (TAPSE) or tricuspid annular peak systolic tissue Doppler velocity (TASV)) to the RV systolic pressure (RVSP) or to the PA systolic pressure [16,17]. Studies evaluating the TAPSE/RVSP ratio in patients with cardiovascular disease have shown associations between a lower TAPSE/RVSP ratio (reflecting worse RV–PA coupling) and adverse outcomes [18,19]. The role of right ventricular function and RV–PA coupling in the prognosis and prediction of clinical outcomes concerning the broad spectrum of cardiovascular diseases needs to be established through current and future studies. Impaired RV–PA coupling was highlighted as a prognostic factor of poor outcomes and increased mortality in critically ill patients hospitalized in a cardiac intensive care unit [20].

Therefore, in this study, we evaluate the role of right ventricular arterial coupling (RVAC) in heart failure symptoms and the functional capacity of patients with HCM.

## 2. Materials and Methods

### 2.1. Study Population

In this cohort study, we included 43 subjects with HCM. The patients we recruited were evaluated in the Unit for Inherited and Rare Cardiovascular Diseases of the First Cardiology Department in Hippokration Hospital between 1 January 2019 and 31 December 2021. We excluded patients who did not have available data to calculate the TASV/RVSP ratio. According to functional capacity and symptoms presentation, patients were categorized according to New York Heart Association (NYHA) classification [21,22]. Patients with normal functional capacity and presentation of shortness of breath at only vigorous activity (NYHA I) were categorized into the asymptomatic group and the rest into the symptomatic group. At the time of the examination, vital signs were obtained, including heart rate and systolic and diastolic blood pressure, and also sudden cardiac death (SCD) risk score was evaluated with the European Society of Cardiology SCD risk score, incorporating data regarding age, maximum LV wall thickness, left atrial size, maximum left ventricular outflow tract gradient, family history of sudden cardiac death, recorded non-sustained ventricular tachycardia, and unexplained syncope [23].

### 2.2. Functional Capacity Assessment

The functional capacity of the enrolled subjects was assessed additionally to NYHA functional classification by means of the six-minute walk test (6MWT). The test was performed according to the American Thoracic Society guidelines in an indoor flat corridor [24]. During the test, subjects were verbally enforced to cover the longest possible distance. For the purpose of this study, the distance in meters covered by each subject during the 6 min period was used as a surrogate measure of its functional status and cardiorespiratory fitness. Based on the median value of the 6MWT distance, the study population was arbitrarily divided into two groups. Those with a 6MWT distance above the median value were characterized as the group of higher 6MWT performance and the rest as the group of lower 6MWT performance. 

### 2.3. Echocardiography

Transthoracic echocardiography (TTE) with the standard protocol was performed. The extent of LV hypertrophy was assessed by measuring the LV wall with the maximum thickness (in mm) measured at any echocardiographic view. The LV ejection fraction (LVEF) was calculated using the Simpson biplane method. LA diameter was measured in the parasternal long axis (PLAX) view. LA dilatation was considered if the LA diameter index to body surface area (BSA) was higher than 2.3 cm/m^2^ [25]. The LVOT maximum gradient was evaluated in the four-chamber echocardiographic view using the pulse wave velocity Doppler at rest and during the Valsalva maneuver. Left ventricular diastolic function was assessed combining mitral inflow pattern (E to A ratio and deceleration time) and mitral annular velocities (e’, measured at the septal and lateral aspects of the mitral annulus in the apical four-chamber view) in combination with left atrial volume index and maximum velocity of the tricuspid regurgitation jet. Additionally, as an index of LV filling pressure, the mitral E/e’ (septal, lateral, and mean) was calculated. The right atrial pressure (RAP) was estimated on the basis of the size and collapsibility of the inferior vena cava. The RVSP was estimated as follows: RAP + [4 × (peak TR velocity)2], based on spectral Doppler. The echocardiographic assessment of the RVAC was made using the TASV/RVSP ratio, which was calculated as the ratio of TASV by tissue Doppler imaging (in cm/s)/the RVSP (in mm Hg). The TASV/RVSP ratio was used to evaluate the right ventricular ability to compensate for the increased afterload [17]. Although several echocardiographic studies have evaluated the adverse prognostic significance of low TAPSE to RVSP ratio in cardiovascular patients [26], the prognostic significance of TASV/RVSP ratio has mainly been established based on invasive assessment of pulmonary artery systolic pressure [27]. Recently, several studies have evaluated and established the performance of the TASV/RVSP ratio using transthoracic Doppler evaluation of RVSP in several conditions [20,28,29].

### 2.4. Statistical Analysis

Continuous variables were tested with the Kolmogorov–Smirnov test and visual inspection of P-P plots for normality of distribution. Accordingly, they were presented as mean with standard deviation or median with 25th and 75th quartile as appropriate. Percentages were used to present categorical variables. Continuous variables were tested for differences between studied groups according to the *t*-test or the Mann–Whitney U test, depending on the normality of their distribution. Differences in categorical variables across the groups were calculated by the formation of contingency tables and the performance of the χ^2^ test. To examine how the examined variables impact the functional classification independently of other possible confounders, we proceeded to a backward logistic regression model. Pearson correlation coefficient was applied to test how RVAC was associated with several parameters. All reported *p*-values were based on two-sided hypotheses. When the *p*-value was less than 0.05, differences were considered statistically significant. Power analysis with an estimated clinically meaningful effect size of 0.9 and a type I error probability of 5% revealed that a sample size of 21 subjects for each group could achieve a power of 80% to reveal differences. All statistical calculations were performed in SPSS software (version 27.0; SPSS Inc., Chicago, IL, USA) and the power analysis with G*power version 3.1.9.6.

## 3. Results

### 3.1. Characteristics of the Study Population

The mean age of the study population was 54 ± 14 years, and 40% were female. In the majority of the study population, the LVEF was >50%. The LA was dilated in 44% of the study population. Almost half (51%) of our study population present symptoms of heart failure and were categorized in the symptomatic group—NYHA 2 or higher. In the symptomatic group, most patients [17] were categorized in NYHA class 2, and there was no patient categorized in NYHA class 4.

### 3.2. Characteristics of the Study Population According to NYHA Classification

The characteristics of our study population according to functional classification are presented in Table 1. Symptomatic patients were at a mean of 13 years older than the asymptomatic group. The representation of the female gender was greater in the symptomatic group compared with the asymptomatic group (54% vs. 24%, *p* = 0.04). Maximum LVOT gradient (46 ± 45 mmHg vs. 17 ± 16 mmHg, *p* = 0.03), RVSP (38 ± 13 mmHg vs. 28 ± 5 mmHg, *p* = 0.02), and the ratio of E/e’ (15 ± 6 vs. 11 ± 4, *p* = 0.03) were higher in the symptomatic group compared with the asymptomatic group. TASV (11 ± 1 cm/sec vs. 13 ± 2 cm/sec, *p* = 0.04) and the RVAC (0.32 ± 0.09 vs. 0.46 ± 0.11, *p* < 0.001) were lower in the symptomatic group compared with the asymptomatic group. However, there was no difference in other potentially influential factors, such as heart rate or systemic blood pressure. Moreover, the SCD risk score did not differ between the two studied groups.

To examine which of the factors revealed to be significant at first were determinants of impaired NYHA classification independently of the impact of other significant covariates, we proceeded to a backward logistic regression analysis which revealed that only age and RVAC were associated with functional capacity, as shown in Table 2. The same was also true when the LVOT gradient > 30 mmHg as a categorical variable was inserted into the model. 

### 3.3. Characteristics of the Study Population According to 6MWT

The median value of the 6 MWT distance was 550 m, and, accordingly, subjects were categorized into the group of higher 6MWT performance and the group of lower 6MWT performance (Table 3). Those with higher 6MWT performance were younger, with a higher prevalence of the male sex, lower LVOT maximum gradient, lower RVSP, and higher RVAC. 

### 3.4. Parameters Associated with Right Ventriculoarterial Coupling

**Figure 1:**
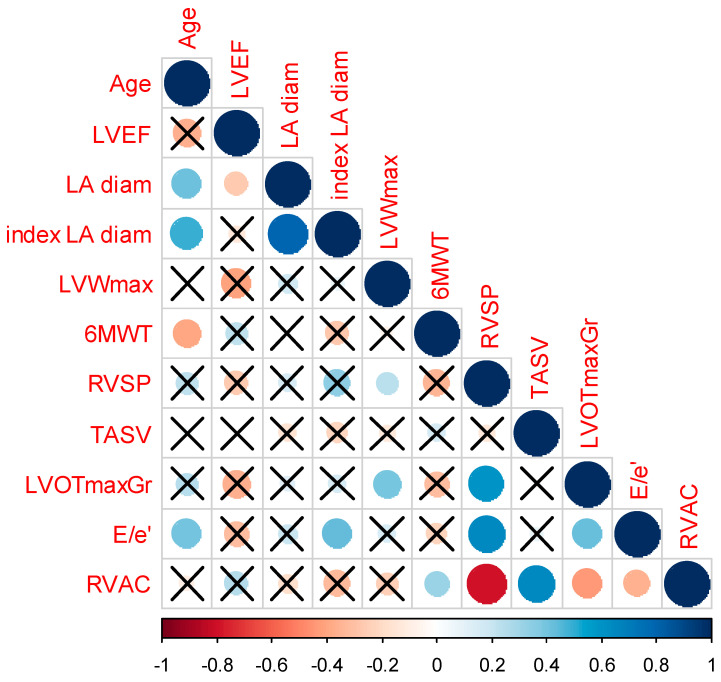
Correlogram expressing the association of RVAC with several variables. The size of the circle expresses the correlation coefficient and the color of the circle expresses the direction of the correlation with scales of red expressing negative association and with scales of blue expressing positive associations. X represents correlations with no statistical significance. Abbreviations: LVEF: left ventricular ejection fraction, LA diam: left atrial diameter, LVWmax: left ventricular maximum wall thickness, 6MWT: six-minute walk test, RVSP: right ventricular systolic pressure, TASV: tricuspid annular peak systolic tissue Doppler velocity, LVOTmaxGr: left ventricular outflow tract maximum gradient, RVAC: right ventriculo–arterial coupling.

To examine which parameters were associated with RVAC, we proceeded to correlations with several variables. It was found that RVAC was positively associated with 6MWT distance and TASV, while it was negatively associated with RVSP, LVOT maximum gradient, and the ratio of e to e prime (Figure 1, Table 4).

## 4. Discussion

To our knowledge, this is the first study examining the role of RVAC, estimated by TTE, in symptoms of patients with HCM independently of LVOT obstruction. Our study showed that the female gender was highly represented in the symptomatic HCM group. Concerning the relationship between the factor of age in patients’ symptoms, symptomatic patients were a mean of 13 years older than the asymptomatic group. Moreover, the echocardiographic measurements of maximum LVOT gradient, RVSP, and the ratio of E/e’ were higher in the symptomatic group compared with the asymptomatic group. Inversely, TASV and RVAC (as TASV/RVSP ratio) had lower values in the symptomatic group, while from the backward logistic regression analysis, it was revealed that age and RVAC were associated with functional capacity independently of the impact of other significant covariates. Concerning the impact of several factors in RVAC in patients with HCM, we found that there was an association between the E/e’ ratio and the LVOT maximum gradient but not with age, sex, or LVEF. 

The role of RVAC and its prognostic significance has already been indicated in PH [30,31], heart failure [26], and dilated cardiomyopathy [32]. Research in patients with heart failure with preserved EF (HFpEF) indicated that TAPSE/RVSP ratio was inversely correlated with NYHA functional class and could also have a role as an independent predictor of adverse outcomes [19]. In HCM, the non-invasive marker of TAPSE/RVSP measured during exercise stress echocardiography in patients without LVOT obstruction was shown to have a significant role in the risk stratification of non-obstructive HCM [33]. The impairment of TAPSE and TAPSE/RVSP ratio during exercise stress echocardiography was correlated with cardiovascular events in these patients [33]. Understanding the role and possible prognostic implications of RV–PA coupling in the clinical entity of HCM may help clinicians to understand the complex interactions between the ventricles and the arterial system in HCM and optimize management strategies accordingly. Interestingly, symptoms, such as fatigue, in patients with HCM and restrictive physiology may be driven by right ventricular dysfunction and incompetence of the right ventricle to increase its cardiac output as per left ventricular preload requirements [34]. 

It is known that patients with HCM have a clinical presentation within a broad spectrum, from being asymptomatic to severely symptomatic requiring hospitalization and sometimes septal reduction therapies [35]. HCM is classified in the two known variants according to the presence of LVOT obstruction as obstructive and non-obstructive. However, symptoms may be present even in patients with the non-obstructive phenotype of HCM [36]. The severity of HCM is conventionally determined either by the degree of LVOT obstruction, generally a consequence of interventricular septum thickening and mitral valve apparatus involvement, or LV aneurysm formation, or the burden of myocardial fibrosis and the sequential myocardial stiffening and diastolic dysfunction [35]. LV diastolic dysfunction and increased LV end-diastolic pressure in HCM are attributed to left ventricular hypertrophy and fibrosis [37]. Remodeling in the extracellular matrix composition in HCM and myocardial fibers disarray also contributes to passive diastolic dysfunction [38,39]. Therefore, left atrial enlargement may be considered a response to chronic pressure and volume overload rather than a direct measure of LV end-diastolic pressure [40]. 

Since HCM is a disease that mainly affects the LV, we could generally mention that the right ventricle and its function is the neglected one. PH is defined in the recently published ESC guidelines as mean pulmonary artery pressure (mPAP) ≥ 20 mmHg [31]. PH, isolated post-capillary or combined with a pre-capillary component, is a frequent consequence of HFpEF, affecting at least 50% of these patients [41]. The presence of severe LVOT obstruction, LV myocardial fibrosis, and LV diastolic dysfunction can lead to the increase in pressures of the pulmonary vasculature and, as a consequence, can cause pulmonary arterial remodeling with an increase in RV afterload [42]. The existence of PH in HCM patients is already known, but its clinical significance, pathophysiology, and impact on the progression of the disease have been recently uncovered [42]. The RV, although not primarily affected in HCM, can also undergo changes. The altered geometry and function of the LV can indirectly influence the right ventricular performance. In HCM, the RV may experience increased afterload due to elevated pulmonary pressures or impaired relaxation of the LV, leading to increased filling pressures and reduced compliance of the RV. The RV–PA coupling, therefore, plays a role in HCM by influencing the overall cardiac function. A disrupted coupling between the RV and the arterial system can further impair ventricular function and lead to symptoms such as exercise intolerance, dyspnea (shortness of breath), and decreased exercise capacity in HCM patients. Examining the prognostic significance of the RV function in patients with HCM, previous research has reported the effect of abnormal RV function on poor outcomes in these patients [43]. In particular, researchers, among other parameters, examined the RV function in HCM patients by measuring the RV four-chamber longitudinal strain via speckle-tracking echocardiography [43]. It was shown that RV dysfunction with impaired RV four-chamber longitudinal strain was associated with adverse outcomes (all-cause mortality and heart failure development) [43]. The role of RV function in the prognosis of poor outcomes, and thus in risk stratification, in patients with HCM needs to be better clarified and established by future research [44]. Furthermore, the RV ventriculo–arterial coupling describes the transfer of energy, via forward blood motion, between the RV and the pulmonary circulation [45]. When afterload increases, pulmonary arterial and then RV elastance must increase to preserve energy transfer through the circulation. If the afterload continues to increase, then the stiffening of the ventricle can lead to reduced RV function. As a result, the RV elastance fails to match increased pulmonary arterial elastance, and thus the RV uncouples from the pulmonary vascular system. Echocardiographic measures of RV function include TAPSE, RV fractional area change (RV-FAC), RV free-wall strain, and TASV. In the recent ESC Guidelines on PH, the TAPSE/RVSP ratio has been offered as a non-invasive measure of RV–PA coupling [31]. In a recently published study, the TASV was measured instead of TAPSE for the calculation of RVAC in patients hospitalized in the cardiac intensive care unit, because TASV is potentially less load dependent than TAPSE [20]. Importantly, such indexes, which are dependent on both the numerator and the denominator, may have an improved ability to identify less significant changes in the physiologic parameters. In our study, we tried to identify the special characteristics of patients with HCM that are identified in symptomatic vs. asymptomatic patients. Concerning patients’ characteristics that have been studied, emphasis was given to echocardiographic, demographic, and general features such as resting heart rate and blood pressure values. As far as we are concerned, our study is the first that has evaluated RVAC, using the measurement of TASV/RVSP, in patients with HCM and correlated these echocardiographic markers with patients’ functional status. Our results demonstrate that TASV/RVSP ratio is an independent determinant of the functional status of HCM patients independently of the presence of LVOT gradient. However, our study, based on its design, has inherent limitations and, importantly, the associations revealed cannot be interpreted as a cause-and-effect association. Furthermore, due to the relatively small sample size, no further sub-analysis can be performed according to the severity of symptoms, the NYHA classification, or the LVOT gradient. Additionally, and beyond the adequate power of our study to detect differences regarding the main outcome, the ability of this analysis to detect secondary associations is limited. Moreover, the right ventricular diastolic function has not been evaluated, posing a significant limitation on the interpretation of our results. 

## 5. Conclusions

This cohort study has pointed out the role of right ventricular function and right ventriculo–arterial coupling in the functional capacity of patients with hypertrophic cardiomyopathy. It is the first study in which the non-invasive echocardiography measurement of the TASV/RVSP ratio was evaluated as an indicator of right ventriculo–arterial coupling in patients with hypertrophic cardiomyopathy and was independently associated with the functional status of these patients. Moreover, right ventriculo–arterial coupling was found to be associated with E/e’ ratio and left ventricular outflow tract maximum gradient but not with age, sex, or left ventricular ejection fraction. Further study is required aiming to examine the possible role of right ventriculo–arterial coupling in risk stratification of patients with hypertrophic cardiomyopathy.

## Figures and Tables

**Table 1:** Clinical and demographic characteristics of the study population.

	Study Population (*n* = 43)	Asymptomatic/NYHA 1 Group (*n* = 21)	Symptomatic/NYHA 2 or Higher Group (*n* = 22)	*p*-Value
Age (years)	54 ± 15	47 ± 16	60 ± 12	0.005
Female gender (%)	40	24	54	0.04
LVEF (%)	59 ± 5	60 ± 5	58 ± 6	0.10
LA diam (mm)	44 ± 6	43 ± 7	46 ± 6	0.14
Index LA diam (mm/m^2^)	23 ± 3	22 ± 3	23 ± 3	0.11
Dilated LA	44	38	50	0.44
LV wall maximum thickness (mm)	18 ± 4	17 ± 2	18 ± 6	0.23
LVOT maximum gradient (mmHg)	32 ± 30	17 ± 16	46 ± 45	0.03
LVOT maximum gradient > 30 mmHg (%)	42	28	65	0.02
RVSP (mmHg)	33 ± 11	28 ± 5	38 ± 13	0.02
TASV (cm/s)	12 ± 2	13 ± 2	11 ± 1	0.04
E/e’	13 ± 6	11 ± 4	15 ± 6	0.03
RVAC	0.40 ± 0.12	0.46 ± 0.11	0.32 ± 0.09	<0.001
SCD risk score (%)	2.74 (1.67, 4.28)	1.79 (1.40, 2.86)	3.67 (2.56, 4.47)	0.09
Heart rate (bpm)	68 ± 12	65 ± 10	72 ± 13	0.11
Systolic blood pressure (mmHg)	122 ± 15	120 ± 14	124 ± 15	0.36
Diastolic blood pressure (mmHg)	79 ± 8	79 ± 10	79 ± 5	0.90
6MWT (m)	507 ± 112	567 ± 62	427 ± 120	<0.001

LVEF: left ventricle ejection fraction; LA diam: left atrium diameter; LVOT: left ventricular outflow tract; RVSP: right ventricular systolic pressure; TASV: tricuspid annular peak systolic tissue Doppler velocity; RVAC: right ventricular arterial coupling; SCD: sudden cardiac death; 6MWT: six-minute walk test.

**Table 2:** Backward binary logistic regression analysis with NYHA status as the dependent variable.

	Odds Ratio	95% Confidence Interval	*p*-Value
Model 1 (age, gender, LVOT maximum gradient, RVSP, TASV, E/e’ ratio, RVAC)			
Age (years)	1.09	1.007, 1.185	0.03
RVAC (ratio)	3.00 × 10^−5^	3.29 × 10^−10^, 0.27	0.006
Model 2 (age, gender, LVOT gradient > 30 mmHg, RVSP, TASV, E/e’ ratio, RVAC)			
Age (years)	1.09	1.007, 1.185	0.03
RVAC (ratio)	3.00 × 10^−5^	3.29 × 10^−10^, 0.27	0.006

Initial variables inserted in model 1: age, gender, LVOT maximum gradient, RVSP, TASV, E/e’ ratio, RVAC. Initial variables inserted in model 2: age, gender, LVOT gradient > 30 mmHg, RVSP, TASV, E/e’ ratio, RVAC. LVOT: left ventricular outflow tract; RVSP: right ventricular systolic pressure; TASV: tricuspid annular peak systolic tissue Doppler velocity; RVAC: right ventricular arterial coupling.

**Table 3:** Clinical and demographic characteristics of the study population according to 6MWT performance.

	Group of Higher 6MWT Performance	Group of Lower 6MWT Performance	*p*-Value
Age (years)	47 ± 16	59 ± 11	0.01
Female gender (%)	16	67	0.02
LVEF (%)	60 ± 5	59 ± 5	0.73
LA diam (mm)	44 ± 7	43 ± 5	0.71
Index LA diam (mm/m^2^)	22 ± 4	23 ± 3	0.49
Dilated LA	42	48	0.63
LV wall maximum thickness (mm)	17 ± 4	17 ± 5	0.95
LVOT maximum gradient (mmHg)	18 ± 18	37 ± 24	0.02
LVOT maximum gradient >30 mmHg (%)	2	65	0.03
RVSP (mmHg)	29 ± 5	35 ± 10	0.02
TASV (cm/s)	12 ± 2	12 ± 2	0.9
E/e’	11 ± 4	14 ± 6	0.09
RVAC	0.45 ± 0.08	0.37 ± 0.13	0.05
SCD risk score (%)	2.62 (1.48, 4.76)	2.62 (1.60, 3.21)	0.43
Heart rate (bpm)	64 ± 10	73 ± 13	0.10
Systolic blood pressure (mmHg)	122 ± 15	121 ± 13	0.81
Diastolic blood pressure (mmHg)	79 ± 10	79 ± 7	0.96

6MWT: six-minute walk test; LVEF: left ventricle ejection fraction; LA diam: left atrium diameter; LVOT: left ventricular outflow tract; RVSP: right ventricular systolic pressure; TASV: tricuspid annular peak systolic tissue Doppler velocity; E/e’: ratio of early transmitral E wave velocity to e’ early mitral annular velocity; RVAC: right ventricular arterial coupling; SCD: sudden cardiac death.

**Table 4:** Correlation of RVAC with other examined variables.

	Age	LVEF	LV Maximum Wall Thickness	LA Diameter	Index LA Diameter	6MWT	RVSP	TASV	LVOTmaxGr	E/e’
R correlation coefficient	−0.74	−0.29	−0.27	−0.17	−0.27	−0.35	−0.77	−0.63	−0.53	−0.38
*p* value	0.65	0.08	0.09	0.29	0.11	0.04	<0.001	<0.001	0.001	0.02

RVAC: right ventriculo–arterial coupling, LVEF: left ventricular ejection fraction, LA: left atrium, 6MWT: six-minute walk test, RVSP: right ventricular systolic pressure, TASV: tricuspid annular peak systolic tissue Doppler velocity, LVOT: left ventricular outflow tract, Gr: gradient, Max. maximum; E/e’: ratio of early transmitral E wave velocity to e’ early mitral annular velocity.

## Data Availability

The data supporting the findings of this study are available from the corresponding author upon reasonable request.
